# Invasive Acantholytic Ocular Surface Squamous Neoplasia: A Rare Case

**DOI:** 10.7759/cureus.39219

**Published:** 2023-05-19

**Authors:** Avi Sharma, Sachin Daigavane, Arvind Bhake

**Affiliations:** 1 Ophthalmology, Jawaharlal Nehru Medical College, Datta Meghe Institute of Higher Education and Research, Wardha, IND; 2 Pathology, Jawaharlal Nehru Medical College, Datta Meghe Institute of Higher Education and Research, Wardha, IND

**Keywords:** invasive carcinoma, malignancy, histopathology, acantholytic variant, squamous cell carcinoma

## Abstract

Ocular surface squamous neoplasia (OSSN) is a spectrum of intraepithelial and invasive neoplastic lesions of the conjunctiva and cornea. OSSN is a rare but potentially sight-threatening ocular malignancy that can be challenging to diagnose due to its clinical and histopathological resemblance to benign ocular surface lesions. However, OSSN can lead to significant ocular and systemic morbidity, including vision loss and metastasis. Various risk factors have been identified, including ultraviolet radiation exposure, human papillomavirus infection, and immunosuppression. The histopathological analysis of the lesion is of utmost importance in diagnosing and further managing squamous cell carcinoma. The acantholytic variant of squamous cell carcinoma is uncommon. Here, we present the case of a 69-year-old male who presented with an invasive mass of progressive growth on the left eyeball extending into the visual axis. The patient underwent extended enucleation, and a histopathological analysis demonstrated a rare acantholytic variant of squamous cell carcinoma.

## Introduction

An ocular surface squamous neoplasia (OSSN) is a precancerous or cancerous lesion afflicting the conjunctival epithelium and cornea [1]. A variety of dysplasias, squamous cell carcinomas, and carcinomas in situ are included in this classification [2]. OSSN is more commonly prevalent among blacks. Those who live in regions in proximity to the equator and are exposed to more sunlight are more likely to suffer from this condition. The OSSN of the conjunctiva and cornea is estimated to affect 0.13 to 3.5 people in every 100,000 population [3]. There is an association between OSSN and exposure to solar ultraviolet radiation, but human papillomavirus (HPV) and human immunodeficiency virus (HIV) are also believed to play a significant role [4-8]. Squamous cell carcinoma development is associated with several mutations in p53 caused by ultraviolet B radiation [4-8]. Tumors usually develop in the nasal sector of the interpalpebral region. The acantholytic variant of OSSN is a rare subtype of OSSN characterized by intraepithelial and invasive neoplastic lesions with marked acantholysis and atypical keratinocytes [9]. A rare case of invasive acantholytic OSSN is presented here with an emphasis on the importance of histopathological analysis in establishing the diagnosis.

## Case presentation

A 69-year-old male, a farmer by occupation, reported to the department of ophthalmology with the chief complaints of a fleshy mass growing in front of the left eye for the past six months, which had an insidious onset and was gradually progressive in nature. It was associated with pain, blepharospasm, watering, and gradual diminution of vision in the left eye, which reduced to visual acuity of hand movements close to the face.

**Figure 1:**
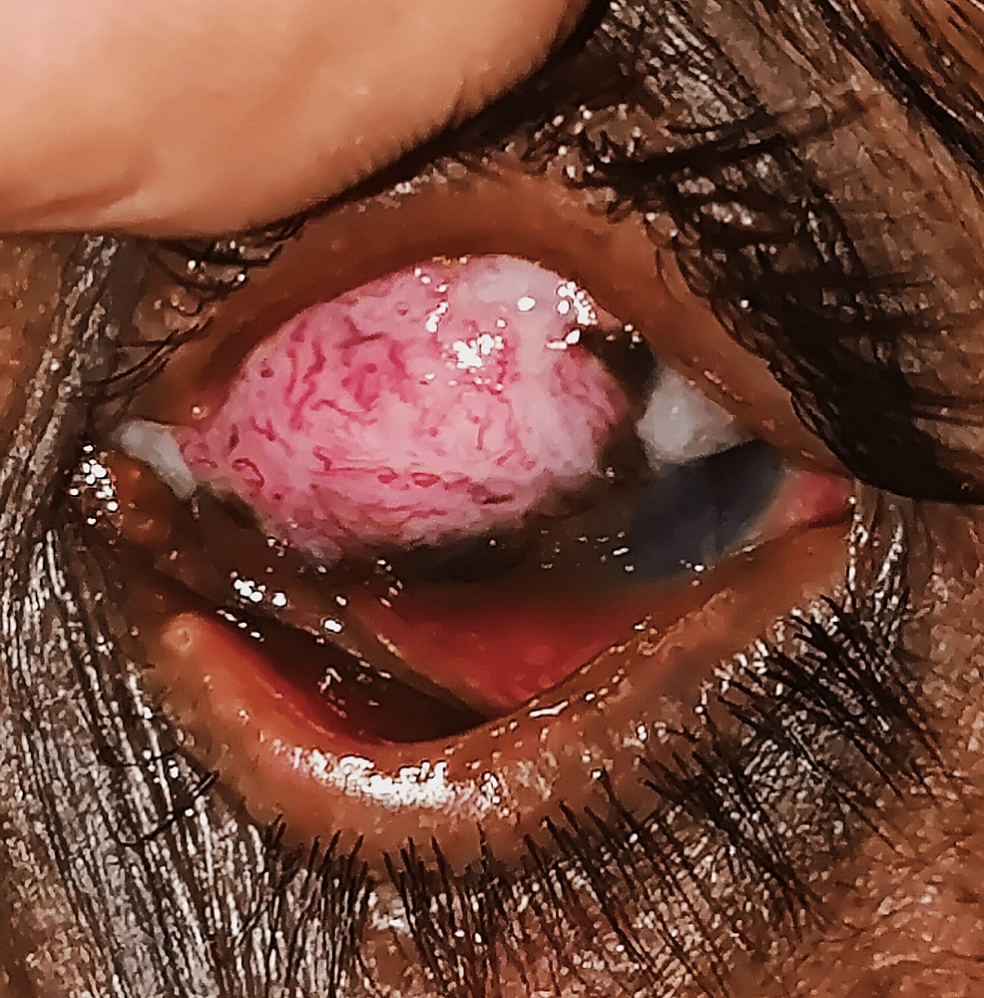
Slit-lamp examination showing a fibrovascular papillomatous conjunctival lesion extending from 7 o’clock to 12 o’clock in the nasal interpalpebral fissure.

On slit-lamp examination, a fibrovascular papillomatous conjunctival lesion extending from 7 o’clock to 12 o’clock in the nasal interpalpebral fissure was observed. The mass encroached the cornea with stromal infiltration encroaching the visual axis. Medial caruncular involvement was evident with surface keratinization and feeder vessels, intrinsic vascularity, and surface pigmentation. The examination of the right eye was normal (Figure 1).

Routine blood testing and serological testing were negative for HIV and HPV. A diagnosis of ocular squamous cell carcinoma was suspected based on the clinical findings, such as a fleshy sessile lesion adjacent to the limbus in the interpalpebral region involving the cornea and bulbar conjunctiva, and the patient underwent extended enucleation with mass excision. The specimen was sent for histopathologic analysis. Postoperatively, the patient was started on topical 1% 5-fluorouracil.

Gross examination of the enucleated eye

**Figure 2:**
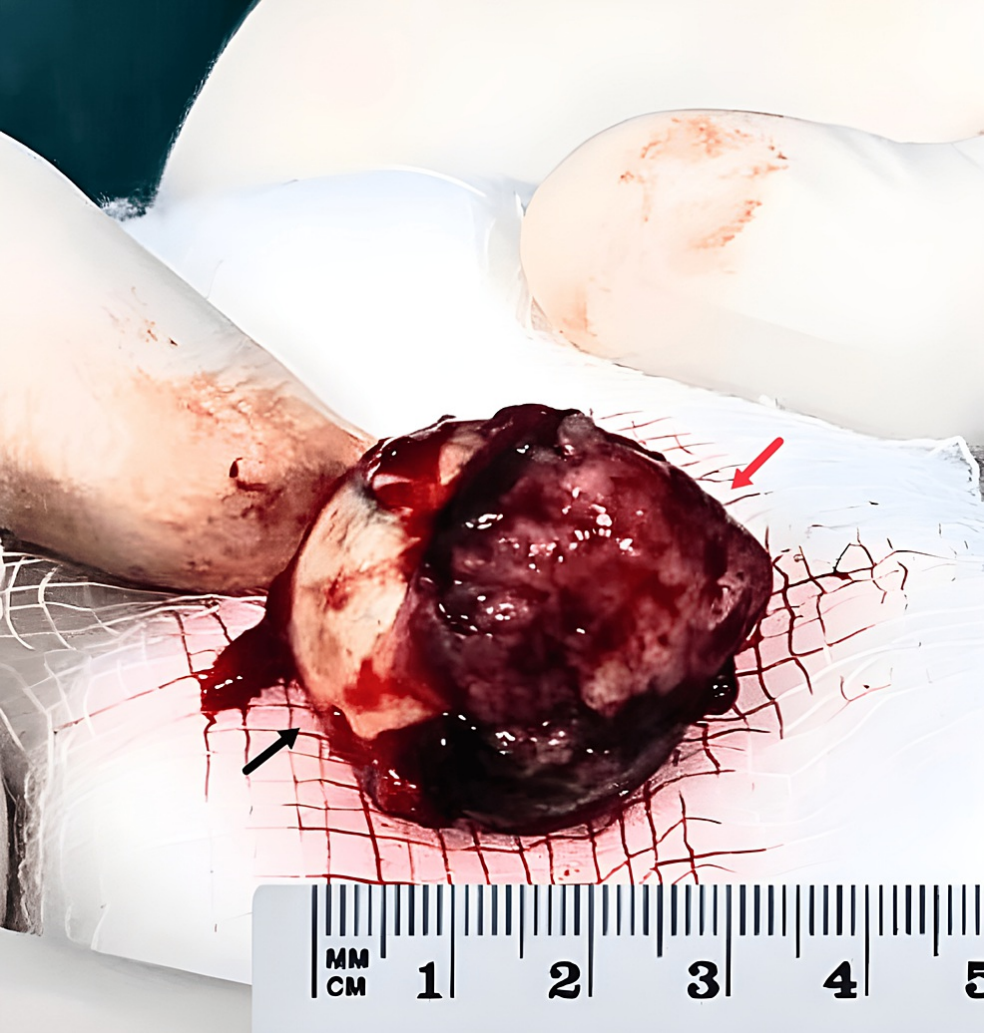
Gross examination of the enucleated specimen. Red arrow: surface growth. Black arrow: eyeball.

An eyeball measuring 3 cm in longest diameter was evaluated with pericorneal and corneal (7 o’clock to 12’o clock) areas which showed apparent growth. The growth measured 1.75 cm × 2 cm, appearing variegated, irregular, and ulcerated. The growth in the part showed a white, fragile, cauliflower-like area. The growth showed no well-defined margins and invaded the cornea, anterior chamber, and sclera on cut section examination. The posterior chamber lacked infiltration by the growth, and the optic nerve stump was free of evident tumor involvement (Figure 2).

Histopathological assessment of the enucleated eye

**Figure 3:**
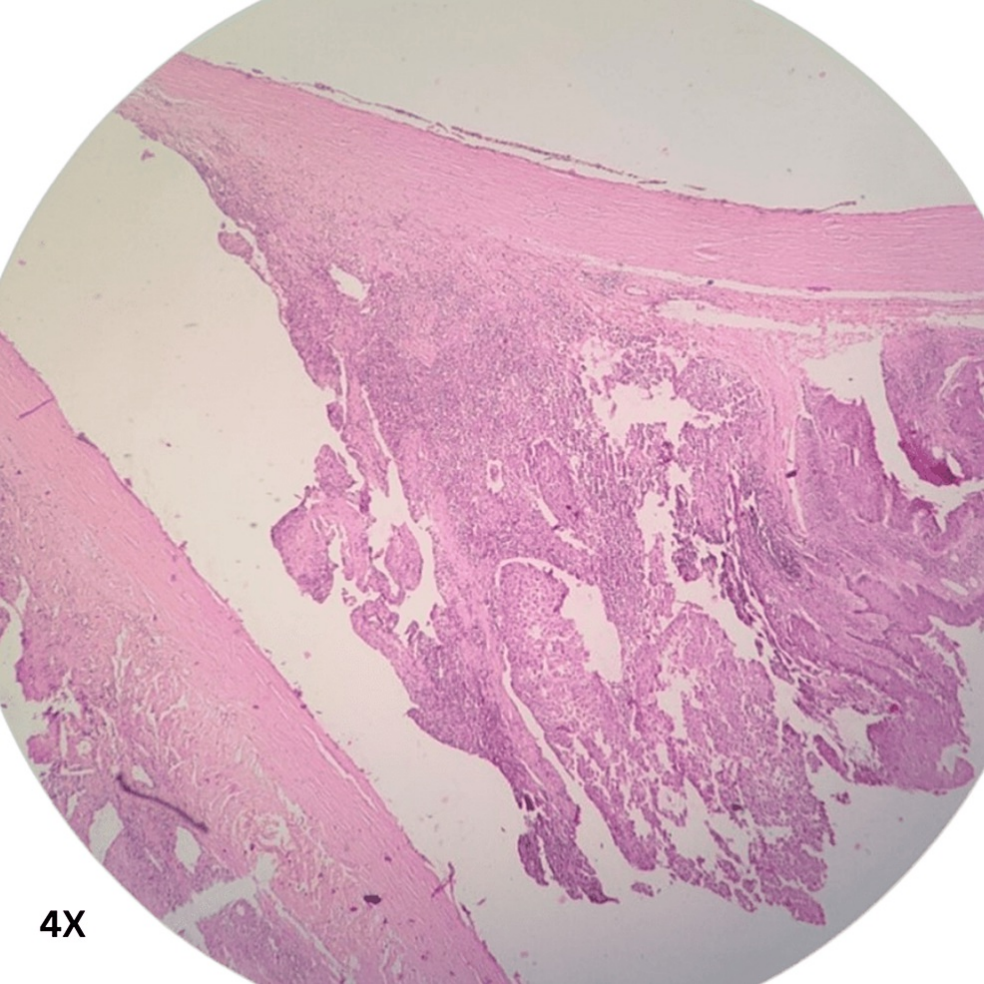
Histo-morphological appearance of an acantholytic variant of ocular squamous cell carcinoma showing papillaroid architecture (magnification 4×).

**Figure 4:**
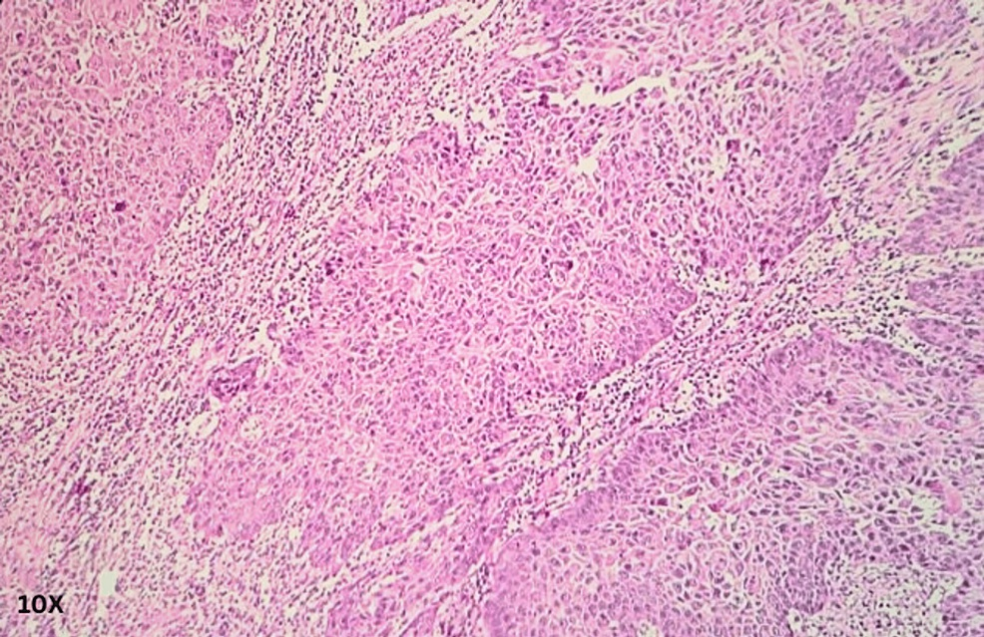
Histo-morphological appearance of an acantholytic variant of ocular squamous cell carcinoma (magnification 10×).

**Figure 5:**
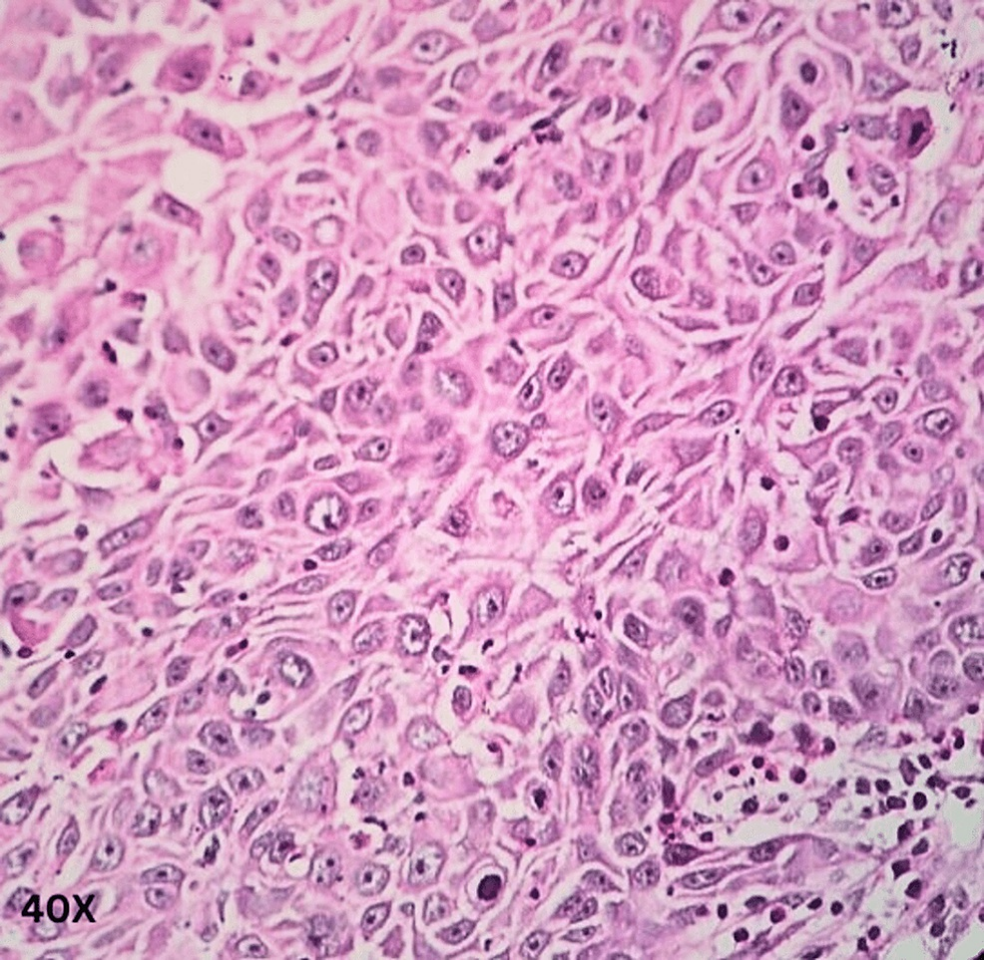
Histo-morphological appearance of an acantholytic variant of ocular squamous cell carcinoma (magnification 40×).

**Figure 6:**
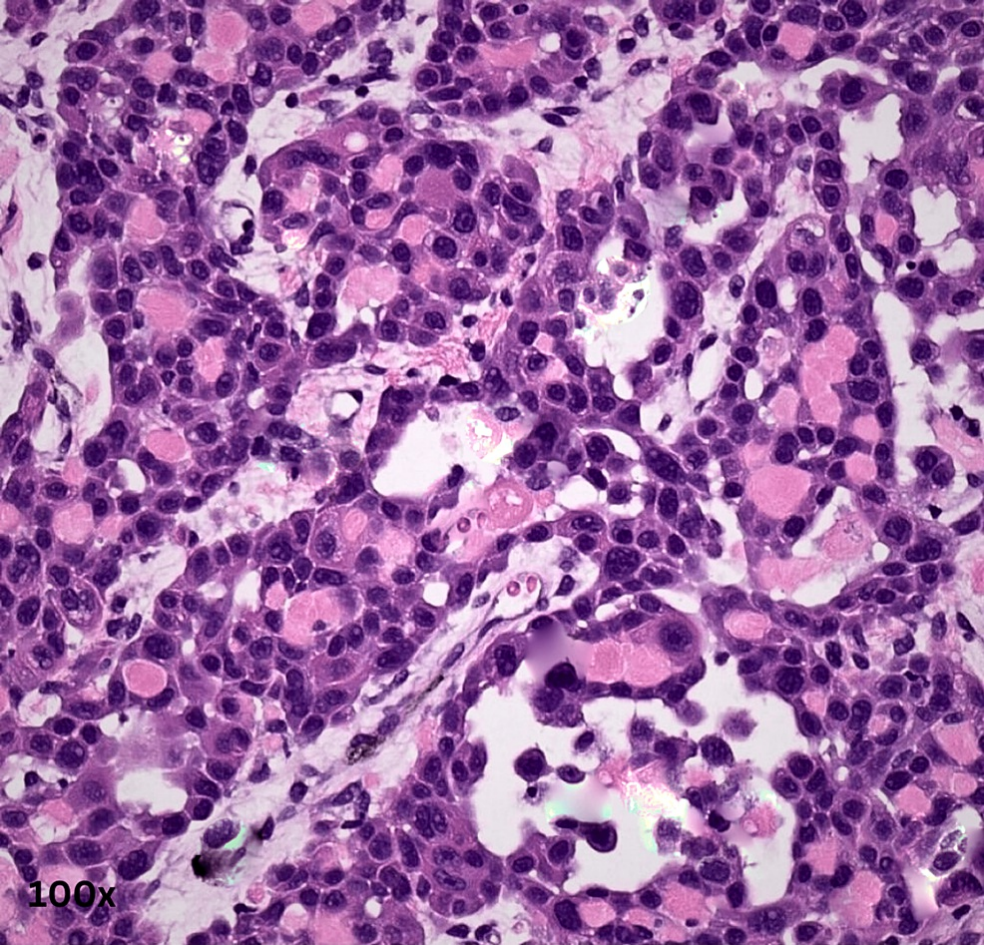
Acantholytic squamous cell carcinoma with multiple acantholytic cells (magnification 100×).

Sections from various areas of the eye and the tumor were taken per the standard reporting protocol. Section from the tumor showed solid sheets, a few finger-like extensions of sheets, some cords, and a pseudo-glandular structure, and the papillaroid structure of squamous cells was noted. The squamous cells contained low-grade malignant nuclei, and their cytoplasm showed partial-to-complete keratinization. The intercellular cracking creating the windows was prominent (acantholytic change). The intercellular bridges were prominent but present in a few cells. No squamous pearls, whorls, or nests were identified. The tumor, at places, showed lymphoid cell infiltrate. The invasion was noticed in scleral tissue below the choroid, but the posterior chamber and optic nerve were free from tumor invasion. A diagnosis of an acantholytic variant of squamous cell carcinoma was offered (Figures 3-6).

Follow-up

**Figure 7:**
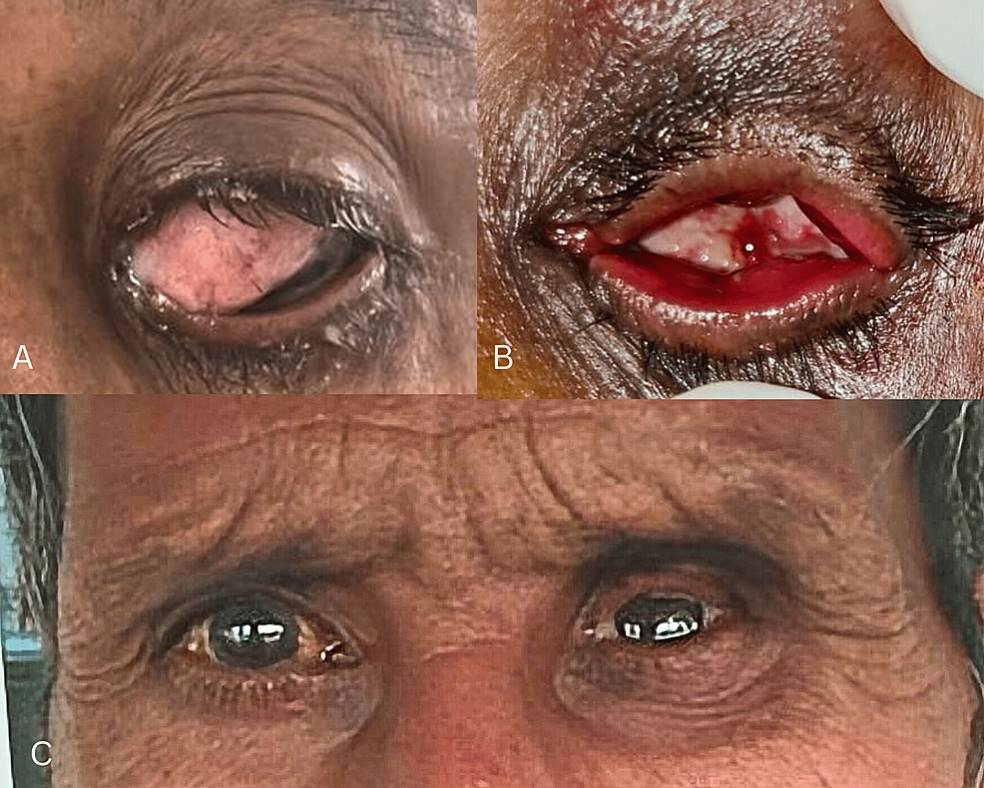
Pre and post-enucleation and orbital prosthesis. Follow-up after one month. A: pre-enucleation. B: immediate postoperative day after extended enucleation. C: After one month of enucleation with an orbital prosthetic.

On the follow-up visit after one month, the orbital socket was healthy, and there was no discharge. The patient was referred to the Department of Prosthodontics, where a clear polymethyl methacrylate ocular prosthetic was designed for the patient (Figures 7A-7C).

## Discussion

In this case report, we examined a case of OSSN, a classification encompassing a range of malignancies affecting the eye surface. These neoplasms include intraepithelial neoplasia as well as invasive squamous cell carcinomas. Early diagnosis is crucial as they can often mimic benign lesions such as pterygiums and chronic conjunctivitis [10]. Other differential diagnosis includes pseudo-epitheliomatous hyperplasia, squamous papilloma, actinic keratosis, pyogenic granuloma, benign epithelial dyskeratosis, chronic inflammation of the ocular surface, corneal pannus, Mooren’s ulcer, corneal epithelial dystrophies and fatty degenerations, keratoacanthoma, conjunctival nevus, and conjunctival malignant melanoma [11-13].

The terminology associated with this condition is varied, including epithelioma, epidermalization, conjunctival and corneal intraepithelial neoplasia, dyskeratosis, Bowen’s disease, and ocular surface epithelial dysplasia [10].

Several factors contribute to the etiology of OSSN, including exposure to chemical carcinogens, ultraviolet radiation, viral infections such as HIV and HPV, and mutations in the p53 tumor suppressor gene [14-16]. Our patient was a farmer at risk for malignancy due to chronic exposure to ultraviolet light. These lesions are commonly found within the interpalpebral fissure, particularly near the limbus, although they may occur at any location on the conjunctiva and cornea [17].

The clinical manifestations of OSSN often involve growth at the limbus and a cluster of blood vessels in the interpalpebral region, similar to our patient who presented with a fibrovascular papillomatous conjunctival lesion in the nasal interpalpebral fissure [5]. However, it is worth noting that the literature rarely reports histological variants, such as acantholytic squamous cell carcinoma. We observed similarities in clinicopathological and histopathological features between our case and the case reported by Julius et al. [18].

OSSN presents with three primary lesion types, namely, gelatinous (papilliform or leukoplakic), nodular, and diffuse [10]. The gelatinous form is characterized by a well-defined sessile papillomatous lesion with dilated conjunctival arteries, while the nodular type appears as elevated mulberry-like masses. On the other hand, the diffuse form is poorly demarcated and exhibits a radial pattern of growth [10]. Our patient presented with a papillomatous vascular mass on the ocular surface.

Histopathologically, OSSN can be classified based on the predominant cell type, such as spindle cell type and mucoepidermoid and adenoid squamous subtypes [10]. High-grade lesions are often associated with male gender, temporal location, multifocality, increased mitotic activity, and low-grade cellular differentiation [19]. Although conjunctival and corneal squamous neoplasia are less likely to undergo systemic metastasis, they can invade local areas.

The acantholytic variant is characterized by histopathological features that involve a non-solid component containing either single or grouped acantholytic and dyskeratotic epithelial cells or cellular debris beneath the conventional squamous cells. This non-solid component is also known to create pseudo-glandular or pseudo-vascular structures [20]. Our histopathological specimen showed solid sheets, finger-like extensions of sheets, cords, a pseudo-glandular structure, and a papillaroid structure of squamous cells. The squamous cells showed intercellular cracking and low-grade malignant nuclei in the form of acantholytic changes. The intercellular bridges were prominent but present in a few cells. Thus, a diagnosis of acantholytic OSSN was made.

Histopathological evaluation is essential to establish a definitive diagnosis and differentiate between the various lesions within the broad spectrum of OSSN. Treatment approaches for primary OSSN have traditionally involved surgery. However, topical chemotherapy is increasingly gaining traction among corneal specialists [20]. A recent study on the standard of care in treating OSSN revealed that while 66% of corneal specialists previously relied solely on surgery for primary OSSN, this percentage decreased to 51% in 2012, with more doctors opting for medical treatments instead [21,22].

It is essential to acknowledge that our findings align with previous reports, further contextualizing our case within the existing literature. This strengthens the evidence supporting the diagnosis and management of OSSN in line with established knowledge. Moreover, our findings contribute to understanding the histological variant of acantholytic squamous cell carcinoma, which has rarely been reported in the literature. By documenting this rare occurrence, we provide valuable information that adds to the body of evidence surrounding OSSN.

## Conclusions

This is a case of a 69-year-old farmer diagnosed with a rare acantholytic variant of invasive ocular squamous cell carcinoma on biopsy. The patient underwent extended enucleation with excision of the mass and was started on topical 5-fluorouracil. Histopathology is the gold-standard method for diagnosing the various types of ocular surface squamous carcinomas. This case report highlights the importance of histopathological analysis in diagnosing and managing rare cases of ocular squamous cell carcinoma. The patient was managed by interdepartmental collaboration between oncology and pathology.
